# Supplementary figures and images for: Systematic review and network meta-analysis on the efficacy and safety of parmacotherapy for hand osteoarthritis
Source: PLoS One. 2024 May 9;19(5):e0298774. doi: 10.1371/journal.pone.0298774 (PMC11081354; doi:10.1371/journal.pone.0298774)

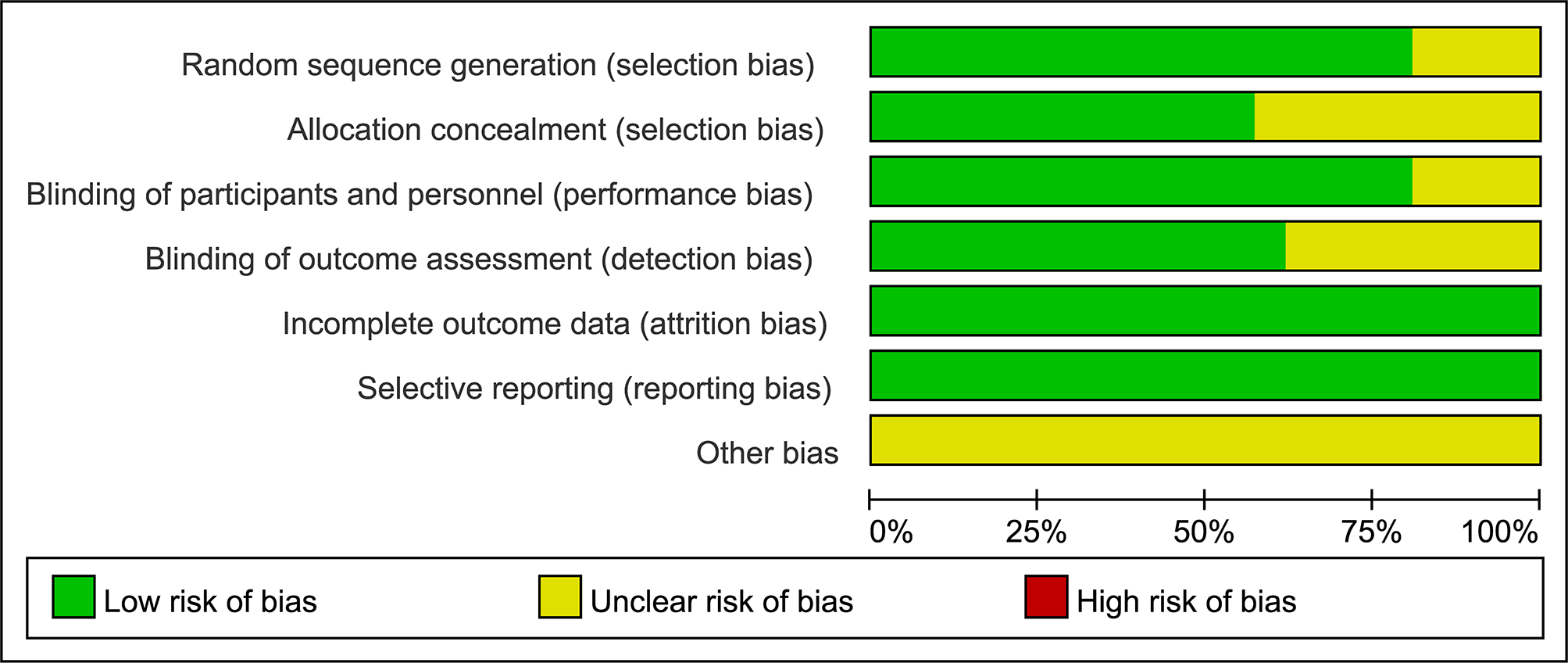

Supplement: S1 Fig — (TIF) [file pone.0298774.s001.tif]

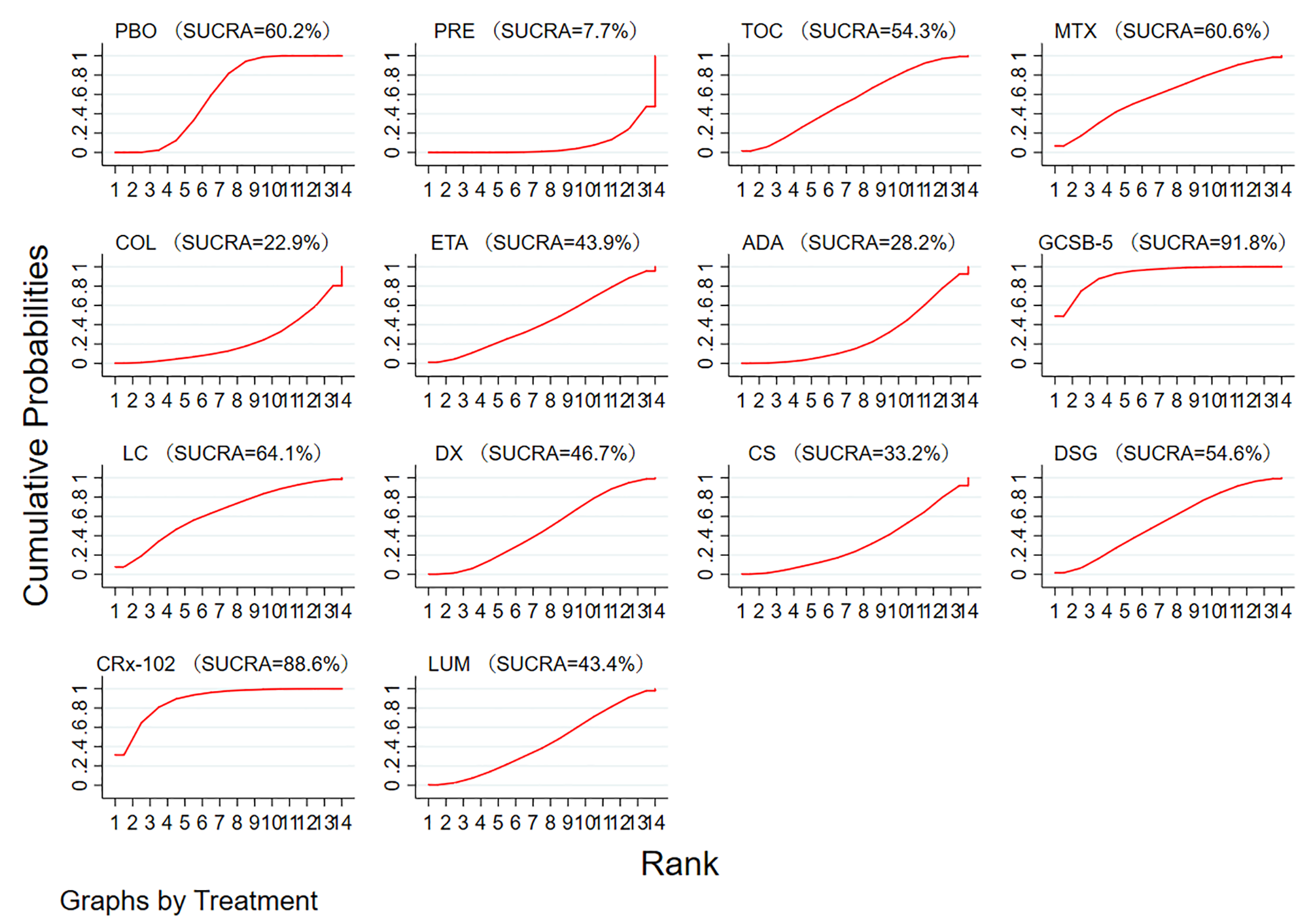

Supplement: S2 Fig — (TIF) [file pone.0298774.s002.tif]

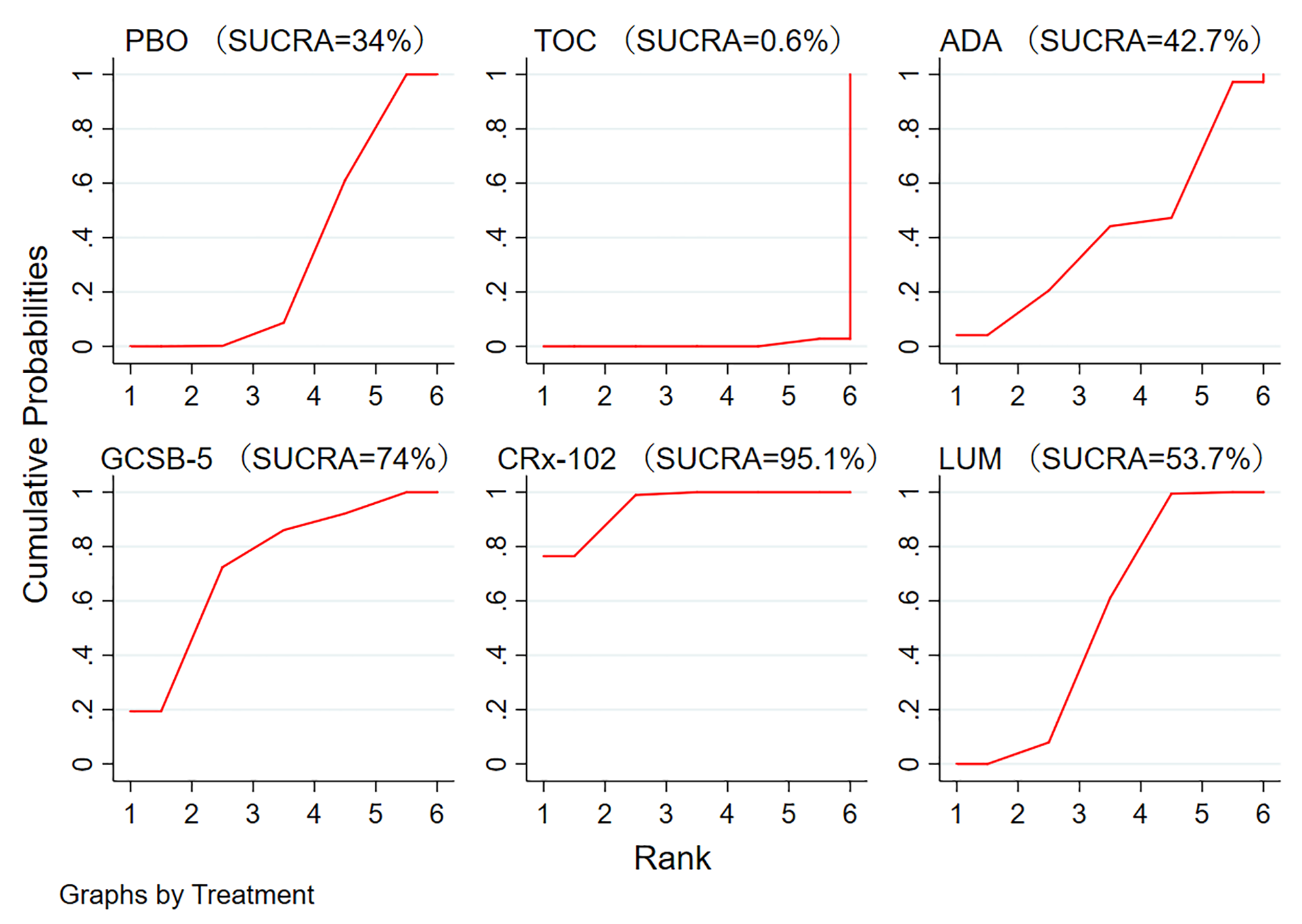

Supplement: S3 Fig — (TIF) [file pone.0298774.s003.tif]

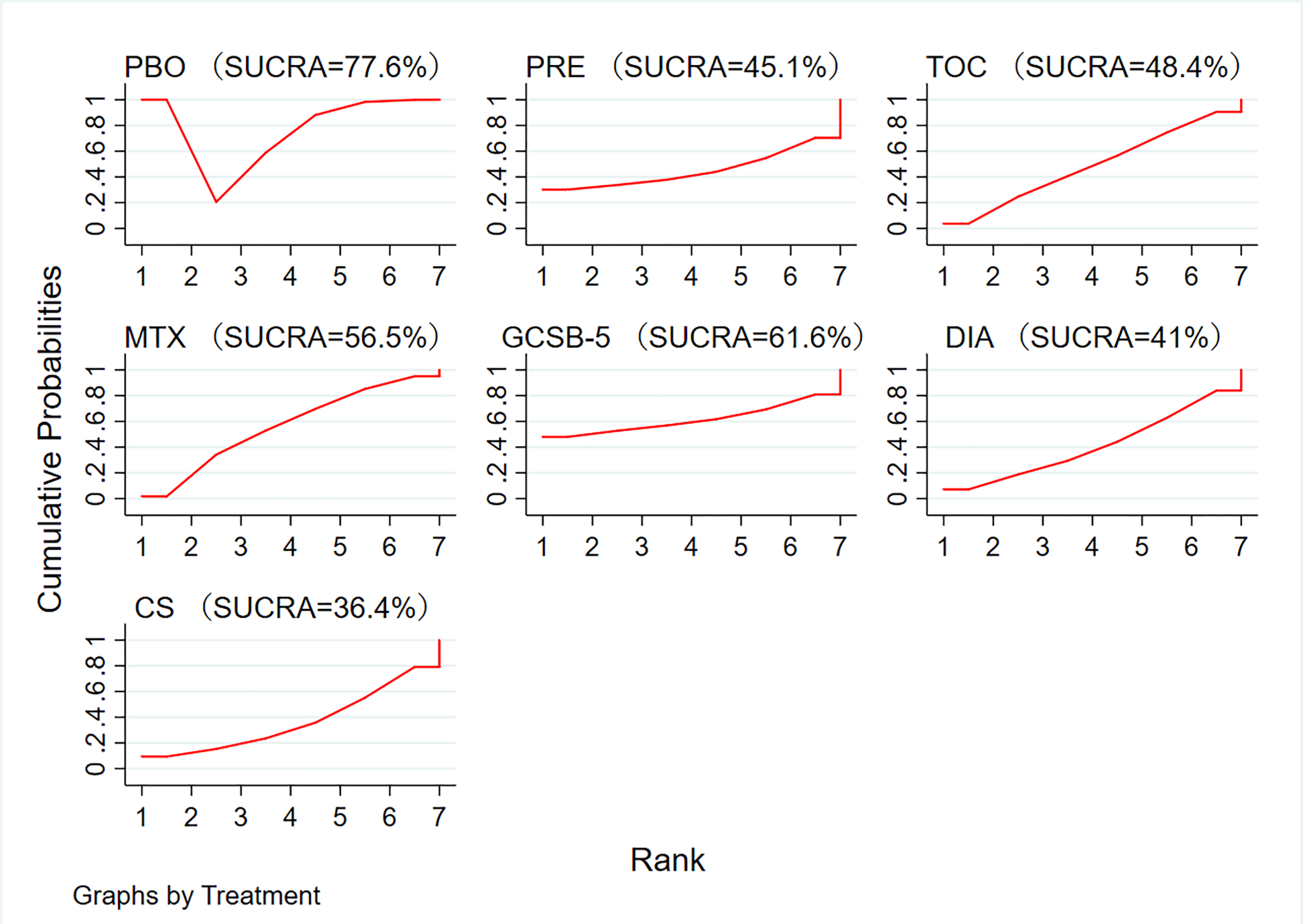

Supplement: S4 Fig — (TIF) [file pone.0298774.s004.tif]

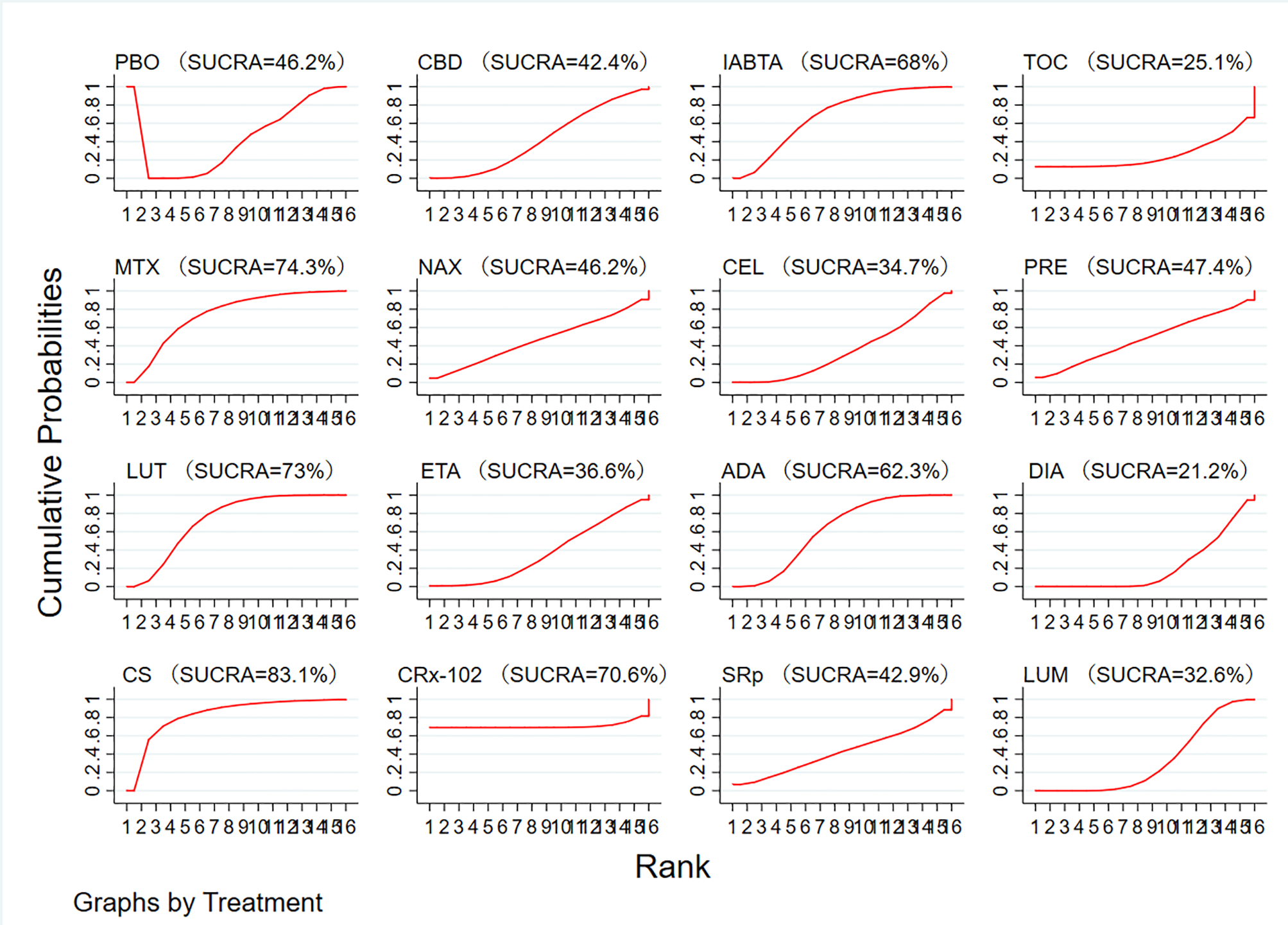

Supplement: S5 Fig — (TIF) [file pone.0298774.s005.tif]

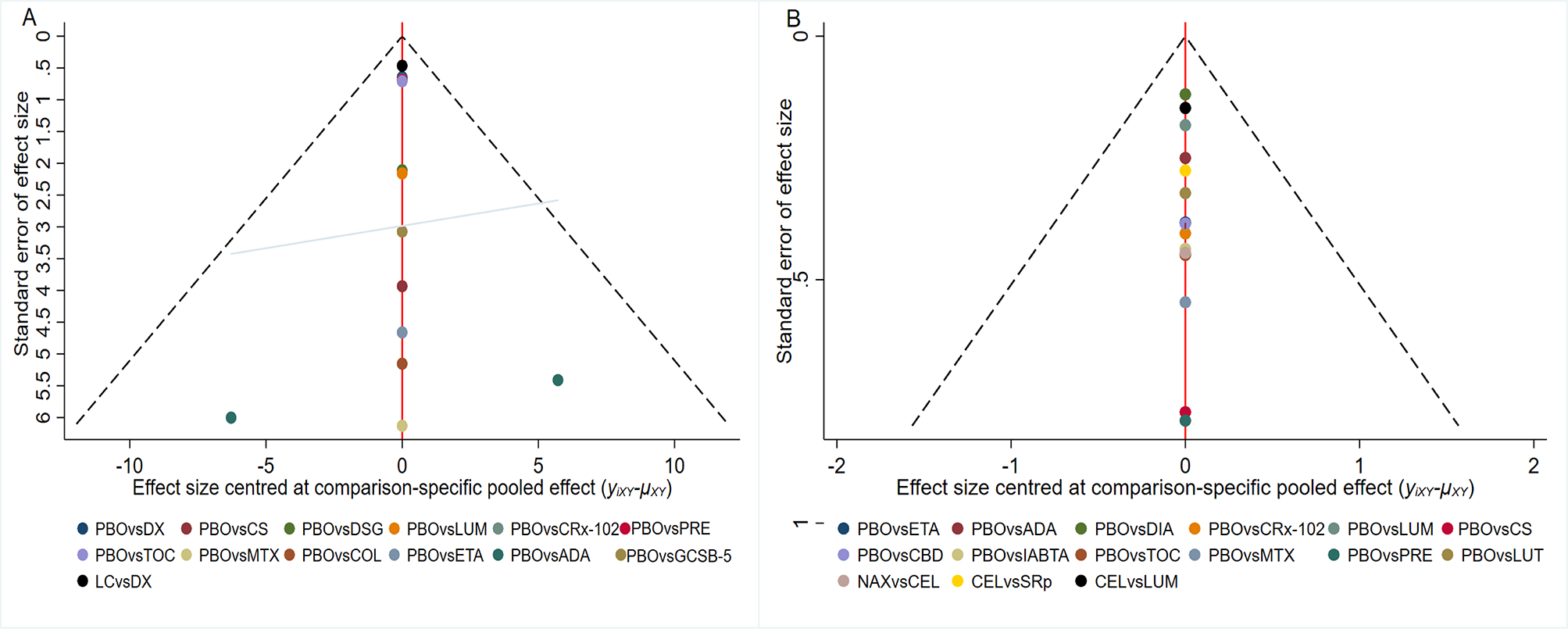

Supplement: S6 Fig — Comparison-correction funnel plot of pain (A) and adverse reaction rate (B). (TIF) [file pone.0298774.s006.tif]
