# Supplementary material for: Systematic review and network meta-analysis on the efficacy and safety of parmacotherapy for hand osteoarthritis
Source: PLoS One. 2024 May 9;19(5):e0298774. doi: 10.1371/journal.pone.0298774 (PMC11081354; doi:10.1371/journal.pone.0298774)
Supplement: S1 Table — (DOCX) [file pone.0298774.s007.docx]

**S1 Table. A** **hierarchy list of data extraction.**

| **Outcome Assessment** | **Rank** |
| --- | --- |
| Pain | (1)VAS global pain score  (2) Pain during activities or at test  (3)WOMAC pain subscale  (4)AUSCAN pain subscale  (5)Other composite pain score  (6)Patients’ global assessment  (7)Physicians’ global assessment |
| Stiffness | (1)WOMAC physical stiffness subscale  (2)AUSCAN physical stiffness subscale  (3)Other composite physical stiffness subscale  (4)patients’ global assessment  (5)physicians’ global assessment |

WOMAC: Western Ontario and McMaster Universities Osteoarthritis Index; AUSCAN: Australian/Canadian Hand Osteoarthritis Index; VAS: visual analogue scale
