# Supplementary material for: Systematic review and network meta-analysis on the efficacy and safety of parmacotherapy for hand osteoarthritis
Source: PLoS One. 2024 May 9;19(5):e0298774. doi: 10.1371/journal.pone.0298774 (PMC11081354; doi:10.1371/journal.pone.0298774)
Supplement: S2 Table — (DOCX) [file pone.0298774.s008.docx]

**S2 Table. Retrieval Strategy in PubMed Database.**

| **Number** | **Search Terms** | **Results** |
| --- | --- | --- |
| #1 | (hand Arthroses [Title/Abstract]) OR (Arthrosis [Title/Abstract]) OR (Degenerative Arthritides [Title/Abstract]) OR (Osteoarthritides [Title/Abstract]) OR (Osteoarthroses [Title/Abstract]) OR (Osteoarthrosis [Title/Abstract]) OR (Osteoarthrosis Deformans [Title/Abstract]) OR (hand OsteoaRthritis [Mesh]) | 13,435 |
| #2 | (Medicine [Title/Abstract]) OR (drug [Title/Abstract]) OR (Medicine [Mesh]) | 3,020,298 |
| #3 | (Glucocorticoid [Title/Abstract]) OR (Glucocorticoid Effect [Title/Abstract]) OR (Glucorticoid Effects [Title/Abstract]) OR (Glucocorticoids [Mesh]) | 106,925 |
| #4 | (Corticosteroids [Title/Abstract]) OR (Corticosteroid [Title/Abstract]) OR (Corticoids [Title/Abstract]) OR (Corticoid [Title/Abstract]) OR (Adrenal Cortex Hormones [Mesh]) | 375,317 |
| #5 | (Amethopterin [Title/Abstract]) OR (Mexate [Title/Abstract]) OR (Methotrexate Sodium [Title/Abstract]) OR (Methotrexate, Disodium Salt [Title/Abstract]) OR (Dicesium Salt Methotrexate[Title/Abstract]) OR (Methotrexate [Mesh]) | 40,957 |
| #6 | (Intra-articular triamcinolone hexacetonide injections[Title/Abstract]) OR (Epidiolex [Title/Abstract]) OR (Cannabidiol [Mesh]) | 3,011 |
| #7 | (Colchicine, (R)-Isomer [Title/Abstract]) OR (Colchicine [Mesh]) | 15,933 |
| #8 | (atlizumab [Title/Abstract]) OR (tocilizumab [Mesh]) | 19 |
| #9 | (Predate [Title/Abstract]) OR (Predonine [Title/Abstract]) OR (Prednisolone [Mesh]) | 55,324 |
| #10 | (TNFR-Fc Fusion Protein [Title/Abstract]) OR (Etanercept [Mesh]) | 6,437 |
| #11 | (Humira [Title/Abstract]) OR (Adalimumab-adbm [Title/Abstract]) OR (Adalimumab [Mesh]) | 6,843 |
| #12 | "randomized controlled trial"[Publication Type] OR "controlled clinical trial"[Publication Type] OR "randomized"[Title/Abstract] OR "randomised"[Title/Abstract] OR "randomization"[Title/Abstract] OR "randomisation"[Title/Abstract] OR "randomly"[Title/Abstract] OR "placebo"[Title/Abstract] OR "trial"[Title/ Abstract] | 1,896,986 |
| #13 | "observational study"[publication type] | 136,077 |
| #14 | #2 OR #3 OR #4 OR #5 OR #6 OR #7 OR #8 OR #9 OR #10 OR #11 | 3,470,799 |
| #15 | #14 AND #1 AND (#12 OR #13) | 332 |
