# Supplementary material for: Systematic review and network meta-analysis on the efficacy and safety of parmacotherapy for hand osteoarthritis
Source: PLoS One. 2024 May 9;19(5):e0298774. doi: 10.1371/journal.pone.0298774 (PMC11081354; doi:10.1371/journal.pone.0298774)
Supplement: S3 Table — (DOCX) [file pone.0298774.s009.docx]

**S3 Table. Retrieval Strategy in Embase Database.**

| **Number** | **Search Terms** | **Results** |
| --- | --- | --- |
| #1 | 'hand osteoarthritis'/exp OR 'hand Arthroses' OR 'Arthrosis' OR 'Degenerative Arthritides' OR 'Osteoarthritides' OR 'Osteoarthroses' OR 'Osteoarthrosis' OR 'Osteoarthrosis Deformans':ti,ab,kw | 14636 |
| #2 | 'medicine'/exp OR 'medicine' OR 'drug' OR 'medicine':ti,ab,kw | 23583342 |
| #3 | 'glucocorticoids'/exp OR 'glucocorticoid' OR 'glucocorticoid effect' OR 'glucorticoid effects':ti,ab,kw | 896176 |
| #4 | 'adrenal cortex hormones'/exp OR 'corticosteroids' OR 'corticosteroid' OR 'corticoids' OR 'corticoid':ti,ab,kw | 1175029 |
| #5 | 'Methotrexate'/exp OR 'Amethopterin' OR 'Mexate' OR 'Methotrexate Sodium' OR 'Methotrexate, Disodium Salt' OR 'Dicesium Salt Methotrexate':ti,ab,kw | 205820 |
| #6 | 'cannabidiol'/exp OR 'intra-articular triamcinolone hexacetonide injections' OR 'epidiolex':ti,ab,kw | 7936 |
| #7 | 'colchicine'/exp OR 'colchicine, (R)-Isomer':ti,ab,kw | 37891 |
| #8 | #2 OR #3 OR #4 OR #5 OR #6 OR #7 | 23747543 |
| #9 | 'atlizumab'/exp OR 'tocilizumab':ti,ab,kw | 24973 |
| #10 | 'prednisolone'/exp OR 'predate' OR 'predonine':ti,ab,kw | 152264 |
| #11 | 'etanercept'/exp OR etanercept:ti,ab,kw OR humira:ti,ab,kw OR 'tnr 001':ti,ab,kw OR 'tnt receptor fusion protein':ti,ab,kw OR 'tnfr-fc fusion protein':ti,ab,kw OR 'tnfr fc fusion pro- tein':ti,ab,kw OR 'tntr-fc':ti,ab,kw OR 'tnr-001':ti,ab,kw OR 'tnr001':ti,ab,kw OR 'etanercept- szzs':ti,ab,kw OR 'tnf receptor type ii-igg fusion pro- tein':ti,ab,kw OR 'tnf receptor type ii igg fusion protein':ti,ab,kw OR 'erelzi':ti,ab,kw OR 'enbrel':ti,ab,kw OR 'recombinant hu- man dimeric tnf receptor type ii-igg fusion protein':ti,ab,kw OR 'recombinant human dimeric tnf receptor type ii igg fusion pro- tein':ti,ab,kw | 38040 |
| #12 | 'adalimumab'/exp OR adalimumab:ti,ab,kw OR  humira:ti,ab,kw OR amjevita:ti,ab,kw OR cyltezo:ti,ab,kw OR  'adalimumab adbm':ti,ab,kw OR 'adalimumab atto':ti,ab,kw | 43616 |
| #13 | antagonist* OR inhibitor* OR blocker* | 2984258 |
| #14 | #9 OR #10 OR #11 OR #12 OR #13 | 3170311 |
| #15 | 'observational study'/exp OR 'non experimental stud- ies':ti,ab,kw OR 'non experimental study':ti,ab,kw OR 'nonexperimental studies':ti,ab,kw OR 'nonexperimental study':ti,ab,kw OR 'observation study':ti,ab,kw OR 'observa- tional studies':ti,ab,kw OR 'observational study':ti,ab,kw | 340013 |
| 16 | 'randomized controlled trial'/exp OR 'randomized controlled trial':ti,ab,it OR 'randomized':ti,ab,it OR 'randomised':ti,ab,it OR 'randomization':ti,ab,it OR 'randomisation':ti,ab,it OR rct:ti,ab,it OR 'randomly':ti,ab,it OR placebo:ti,ab,it | 1761007 |
| 17 | #15 OR #16 | 2072452 |
| 18 | #1 AND #17 | 1168 |
| 19 | #8 OR #14 | 24301382 |
| 20 | #18 AND #19 | 550 |
