# Supplementary material for: Systematic review and network meta-analysis on the efficacy and safety of parmacotherapy for hand osteoarthritis
Source: PLoS One. 2024 May 9;19(5):e0298774. doi: 10.1371/journal.pone.0298774 (PMC11081354; doi:10.1371/journal.pone.0298774)
Supplement: S4 Table — (DOCX) [file pone.0298774.s010.docx]

**S4 Table. Retrieval Strategy in Web of Science Database.**

| **Number** | **Search Terms** | **Results** |
| --- | --- | --- |
| #1 | TS=(hand osteoarthritis OR "hand Arthroses" OR "Arthrosis" OR "Degenerative Arthritides" OR "Osteoarthritides" OR "Osteoarthroses" OR "Osteoarthrosis" OR "Osteoarthrosis Deformans") | 23331 |
| #2 | TS=(Medicine OR “Medicine” OR “Drug” OR “Medicine”) | 18011595 |
| #3 | TS=(Glucocorticoids OR "Glucocorticoid" OR "Glucocorticoid Effect" OR "Glucorticoid Effects") | 198237 |
| #4 | TS=(Adrenal Cortex Hormones OR "Corticosteroids" OR "Corticosteroid" OR "Corticoids" OR "Corticoid") | 251858 |
| #5 | TS=(Methotrexate OR "Amethopterin" OR "Mexate" OR "Methotrexate Sodium" OR "Methotrexate, Disodium Salt" OR "Dicesium Salt Methotrexate") | 114392 |
| #6 | TS=(Cannabidiol OR "Intra-articular triamcinolone hexacetonide injections" OR "Epidiolex") | 10283 |
| #7 | TS=(Colchicine OR "Colchicine, (R)-Isomer") | 38600 |
| #8 | #2 OR #3 OR #4 OR #5 OR #6 OR #7 | 18190886 |
| #9 | (Atlizumab OR "tocilizumab") | 11993 |
| #10 | (Prednisolone OR "Predate" OR "Predonine") | 77806 |
| #11 | TS=(Etanercept OR “TNFR- Fc Fusion Protein” OR “TNFR Fc Fusion Protein” OR “TNR 001” OR “TNT Receptor Fusion Protein” OR “TNTR-Fc” OR “TNR-001” OR “TNR001” OR “Etanercept-szzs” OR “TNF Receptor Type II- IgG Fusion Protein” OR “TNF Receptor Type II IgG Fusion Protein” OR “Erelzi” OR“Enbrel” OR “Recombinant Human Dimeric TNF Receptor Type II- IgG Fusion Protein” OR “Recombinant Human Dimeric TNF Receptor Type II IgG Fusion Protein”) | 20406 |
| #12 | TS=(Adalimumab OR Humira OR Adalimumab-adbm OR Amjevita OR Adalimumabatto OR Cyltezo OR “D2E7 Antibody”) | 23096 |
| #13 | TS=("antagonists and inhibitors" OR "inhibitor" OR "antago-nist" OR "blocker" OR "inhibitors" OR "antagonists" OR "blockers") | 3076439 |
| #14 | #9 OR #10 OR #11 OR #12 OR #13 | 3173957 |
| #15 | TS=(Randomized controlled trial) | 716054 |
| #16 | TS=(observational study) | 334103 |
| #17 | #15 OR #16 | 1024826 |
| #18 | #1 AND #17 AND #14 | 255 |
