# Supplementary material for: Systematic review and network meta-analysis on the efficacy and safety of parmacotherapy for hand osteoarthritis
Source: PLoS One. 2024 May 9;19(5):e0298774. doi: 10.1371/journal.pone.0298774 (PMC11081354; doi:10.1371/journal.pone.0298774)
Supplement: S5 Table — (DOCX) [file pone.0298774.s011.docx]

**S5 Table. Retrieval Strategy in Cochrane Library Database.**

| **Number** | **Search Terms** | **Results** |
| --- | --- | --- |
| #1 | ('hand osteoarthritis' OR 'hand Arthroses' OR 'Arthrosis' OR 'Degenerative Arthritides' OR 'Osteoarthritides' OR 'Osteoarthroses' OR 'Osteoarthrosis' OR 'Osteoarthrosis Deformans'):ti,ab,kw | 2445 |
| #2 | (‘Medicine’ OR 'Medicine’ OR 'Drug' OR 'Medicine'）:ti,ab,kw | 849987 |
| #3 | ('Glucocorticoids' OR 'Glucocorticoid' OR 'Glucocorticoid Effect' OR 'Glucorticoid Effects'):ti,ab,kw | 10037 |
| #4 | ('Adrenal Cortex Hormones' OR 'Corticosteroids' OR 'Corticosteroid' OR 'Corticoids' OR 'Corticoid'):ti,ab,kw | 26777 |
| #5 | ('Amethopterin‘ OR ’Mexate‘ OR ’Methotrexate Sodium‘ OR ’Methotrexate, Disodium Salt‘ OR ’Dicesium Salt Methotrexate‘ OR ’Methotrexate):ti,ab,kw | 12558 |
| #6 | ('Intra-articular triamcinolone hexacetonide injections' OR 'Epidiole' OR 'Cannabidiol):ti,ab,kw | 1071 |
| #7 | (Colchicine‘ OR ‘Colchicine, (R)-Isomer‘):ti,ab,kw | 1163 |
| #8 | #2 OR #3 OR #4 OR #5 OR #6 OR #7 | 864210 |
| #9 | ('Atlizumab' OR 'tocilizumab'):ti,ab,kw | 1530 |
| #10 | ('Predate' OR 'Predonine' OR 'Prednisolone'):ti,ab,kw | 142 |
| #11 | ('Etanercept' OR 'TNFR-Fc Fusion Protein'):ti,ab,kw | 2381 |
| #12 | ('Humira' OR 'Adalimumab-adbm' OR 'Adalimumab'):ti,ab,kw | 470 |
| #13 | ('antagonists and inhibitor' OR 'inhibitor' OR 'antago-nist' OR 'blocker' OR 'inhibitors' OR 'antagonists' OR 'blockers):ti,ab,kw | 130221 |
| #14 | #9 OR #10 OR #11 OR #12 OR #13 | 133585 |
| #15 | ('randomized controlled trial' OR 'randomized' OR 'randomised' OR 'randomization' OR 'randomisation' OR 'rct' OR 'blockers OR 'randomly' OR 'placebos'):ti,ab,kw | 1250544 |
| #16 | #14 OR #15 | 1284745 |
| #17 | #1 AND #16 | 217 |
