# Supplementary material for: Systematic review and network meta-analysis on the efficacy and safety of parmacotherapy for hand osteoarthritis
Source: PLoS One. 2024 May 9;19(5):e0298774. doi: 10.1371/journal.pone.0298774 (PMC11081354; doi:10.1371/journal.pone.0298774)
Supplement: S6 Table — (DOCX) [file pone.0298774.s012.docx]

**S6 Table.** **Occurrence of adverse reactions.**

| First Author, Publication Year | Sample size | | Interventions | | Adverse reactions | |
| --- | --- | --- | --- | --- | --- | --- |
|  | T | C | T | C | T | C |
| Nguyen.etal，2022^[17]^ | 30 | 30 | IABTA | PBO | Base-of-thumb pain: 6; Thumb paraesthesia: 1 | Base-of-thumb pain: 11;Thumb paraesthesia: 1 |
| Kloppenburg.etal，2019（1）^[18]^ | 64 | 67 | LUT | PBO | Injection site reaction: 23; Serious adverse events: 2 | Injection site reaction: 11;Serious adverse events: 2 |
| Richette.etal，2021^[19]^ | 42 | 41 | TOC | PBO | Infections: 12; Serious adverse events: 3 | Infections: 6;Serious adverse events: 1 |
| Ferrero.etal,2021^[20]^ | 32 | 32 | MTX | PBO | Discomfort: 1；Nausea: 1；Abdominal Pain: 1；Diarrhoea: 1 | Pericarditis: 1;Cough: 1;Abdominal Pain: 3;Diarrhoea: 3;Nasal cavity cancer: 1 |
| Park.etal,2020（1）^[22]^ | 52 | 53 | NAX | CE | Gastrointestinal disorders: 3；Infections and infestations: 1；Nervous system disorders: 1；Eye disorders: 1；Skin and subcutaneous tissue disorders: 2 | Gastrointestinal disorders: 1；Infections and infestations: 3；Metabolism and nutrition disorders: 2；Skin and subcutaneous tissue disorders: 3 |
| Kroon.etal,2019^[23]^ | 46 | 46 | PRE | PBO | Gastrointestinal disorders: 3 | Gastrointestinal disorders: 4 |
| Kloppenburg.etal,2018（2）^[24]^ | 45 | 45 | ETA | PBO | Cardiovascular: 1；Skin and subcutaneous tissue disorders: 3；Gastrointestinal disorders: 2；other: 4 | Skin and subcutaneous tissue disorders: 1；Cardiovascular: 4；Gastrointestinal disorders: 2；other: 3 |
| D.Aitken.etal,2018^[25]^ | 18 | 25 | ADA | PBO | Headache: 3；Other joint pain: 2；Upper respiratory tract infection: 1；Vertigo: 2；Increased hand pain/swelling: 2；Nausea: 2；other: 3 | Headache: 5；Other joint pain: 6；Insomnia: 1；Upper respiratory tract infection: 5；Increased hand pain/swelling: 1；Nausea: 1;other: 4 |
| Kichul.etal,2013^[29]^ | 42 | 44 | DIA | PBO | Skin rash: 3；Diarrhea: 9；Nausea: 3；Abdominal pain: 13；Headache: 10； | Diarrhea: 9；Nausea: 2；Abdominal pain: 6；Headache: 12 |
| Gabay.etal,2011^[30]^ | 80 | 82 | CS | PBO | Gastrointestinal disorders: 12 | Gastrointestinal disorders: 14 |
| Kvien.etal,2008^[32]^ | 42 | 41 | CRx-102 | PBO | Headache: 22 | Headache: 6 |
| Grifka.etal,2004^[33]^ | 205 | 196 | LUM | PBO | Gastrointestinal disorders: 15；other: 36 | Gastrointestinal disorders: 13;other: 29 |
| Vela.etal,2022^[35]^ | 70 | 66 | CBD | PBO | Musculoskeletal: 11；Serious adverse events: 2 | Musculoskeletal: 11；Serious adverse events: 2 |
| Yelland.etal,2007^[36]^ | 41 | 41 | CEL | SRp | Dizziness: 6；Nausea: 7；Stomach pains:6 | Dizziness: 6；Nausea: 9；Stomach pains: 11 |
| Fleischmann.etal,2008^[37]^ | 755 | 758 | LUM | CEL | Headache: 82；Dyspepsia: 38 | Headache: 82；Dyspepsia: 39 |
