# Supplementary material for: Systematic review and network meta-analysis on the efficacy and safety of parmacotherapy for hand osteoarthritis
Source: PLoS One. 2024 May 9;19(5):e0298774. doi: 10.1371/journal.pone.0298774 (PMC11081354; doi:10.1371/journal.pone.0298774)
Supplement: Attachment — Submitted filename: comments.docx [file pone.0298774.s014.docx]

The authors have chosen a very nice research area. Thank you for your effort and contribution to science.

N.B. my expertise is on the methodology and it would be difficult to say much on the scientific aspects.

Comments

1. On the title part, it would be better if you included “systematic review and network meta-analysis”.
2. Abstract- line 27 “ Comprehensive”
3. Result – line 35, the confidence interval includes both negative and positive. How does it show a reduction in pain?

- Please describe the abbreviations GCSB-5, CRx-102, and FIHOA?

4. Keyword- it should be a network meta-analysis, not a meta-analysis.
5. Introduction- please change it into three or more paragraphs.
6. Method- please provide a citation for “The PRISMA extension….”

- Where is the description of for geometry of the network?
- Intervention- try to describe all the interventions with their dose.
- Line 149- why did you conduct a conventional meta-analysis? You don’t need to conduct it. Can you explain why you did it?

7. Result –

- Line 172-187- you don’t need to explain all these things. Make the paragraph short and try your figure to explain all those steps.
- Line 216-224- it would be good if you could take this one to the method section. No need to describe it in the result part.
- You don’t need to do a conventional meta-analysis. Please remove it.
- For all the results, please provide us with a forest plot. It would be easier for the reader to understand the result than SCURA ranking.
- For all the results, please provide us with the within and between study heterogeneity (I^2^, p-value, and Q). or your test for inconsistency and heteroscedastic.

8. Try to include more studies in the discussion part. Also, try to make the paragraph shorter. Try also to remove irrelevant descriptions from the discussion. Try to focus on the result you got and what makes it similar or different from other studies.
